# Cardiac safety of chronic inhibition of the myostatin–activin pathway with bimagrumab in healthy older adults

**DOI:** 10.1210/clinem/dgag091

**Published:** 2026-03-25

**Authors:** Daniel Rooks, Denise P Yates, Srikanth Neelakantham, Jens Praestgaard, Ricardo C Cury, Hildo J Lamb, Ronenn Roubenoff, Olivier Petricoul, Estelle Lach-Trifilieff, Eric C Svensson

**Affiliations:** Translational Medicine, Biomedical Research, Novartis, Cambridge, MA 02139, USA; Translational Medicine, Biomedical Research, Novartis, Cambridge, MA 02139, USA; AQS Statistical Programming, Novartis Healthcare Private Limited, Hyderabad 500081, India; IQS, Novartis Pharmaceuticals Corporation, East Hanover, NJ 07936, USA; Miami Cardiac and Vascular Institute, Baptist Health South Florida, Miami, FL 33176, USA; Department of Radiology, Florida International University, Miami, FL 33176, USA; Leiden University Medical Center, Leiden University, Leiden 2311EZ, The Netherlands; Translational Medicine, Biomedical Research, Novartis, Cambridge, MA 02139, USA; Translational Medicine, Biomedical Research, Novartis, 4033 Basel, Switzerland; Diseases of Aging and Regenerative Medicine, Biomedical Research, Novartis, 4033 Basel, Switzerland; Translational Medicine, Biomedical Research, Novartis, Cambridge, MA 02139, USA

**Keywords:** bimagrumab, myostatin–activin pathway, cardiac muscle, cardiovascular safety, older adult

## Abstract

**Context:**

GLP-1 receptor agonists have revolutionized the treatment of obesity and type 2 diabetes, but may cause excess muscle loss. Inhibitors of the myostatin–activin pathway can cause fat loss and skeletal muscle gain, but the effect of chronic pathway inhibition on human cardiac muscle is not known.

**Objective:**

Investigate the effects of extended inhibition of the myostatin–activin pathway on cardiovascular parameters in healthy older adults.

**Design:**

Randomized, double-blind, placebo-controlled study with 6 months of treatment and up to 6 months of follow-up.

**Setting:**

Single commercial study site

**Participants:**

68 healthy community-living men and women aged 60 to 86 years.

**Interventions:**

Intravenous bimagrumab 10 mg/kg or placebo.

**Main outcome measures:**

Cardiac magnetic resonance assessment of changes in left ventricular mass index (LVMI) and left ventricular ejection fraction (LVEF). Changes in total lean body mass (LBM) and total body fat mass (FM) by dual energy X-ray absorptiometry (DXA).

**Results:**

At 6 months, no clinically relevant change was observed in LVMI (least squares mean [90% confidence interval] 1.6 g/m^2^ [−0.2, 3.4], *P* = .148) or LVEF (2.0% [−0.4, 4.4], *P* = .176) between treatments. Total LBM (mean [standard deviation]) increased by 5.5% [3.6], and FM decreased by −14% [8.9] with bimagrumab vs placebo (both *P* < .001).

**Conclusion:**

Six months of myostatin–activin pathway inhibition with bimagrumab had no effect on cardiac structure or function in healthy older adults compared to placebo. These results support consideration of bimagrumab as a skeletal muscle-sparing intervention in adults undergoing weight loss with GLP-1 receptor agonists.

Glucagon-like peptide-1 (GLP-1) receptor agonists, alone or in combination with glucose-dependent insulinotropic peptide (GIP) agonists or antagonists, have revolutionized obesity treatment over the past few years. These and other emerging incretin-related drugs continue to demonstrate substantial weight loss in a variety of patient populations and have changed the way obesity, type 2 diabetes, and other conditions of excessive adiposity are medically managed (1). While GLP-1 receptor agonists show a predictable loss of body weight and adipose tissue, the sizable concomitant loss of skeletal muscle raises concern about inducing muscle weakness, falls, disability, or other undesirable effects, especially in older patients and those with pre-existing low muscle mass due to sarcopenia or chronic disease (2).

An ongoing area of drug development involves biological targets that modulate pathways for skeletal muscle anabolism and adipose tissue catabolism to treat conditions associated with skeletal muscle wasting and metabolic dysfunction (3, 4), and potentially for the treatment of damaged cardiac muscle or heart failure (5-7). One such target is the activin type II receptor (ActRII) and its multiple ligands, which are members of the transforming growth factor-beta (TGFβ) superfamily. These ligands, including myostatin (GDF-8), activin A, and growth and differentiation factor-11 (GDF-11), limit skeletal muscle mass growth through the Smad 2/3 pathway by inhibiting muscle protein synthesis and myocyte differentiation and proliferation.

The ActRII receptor also has direct effects on adipose tissue accretion of fatty acids. The absence of these ligands, primarily myostatin, in developing animals and humans results in a hypermuscular phenotype due to an increased number and size of muscle fibers (8-10). Reducing pathway activity by targeting individual ligands or the receptor directly in humans (11-13) and preclinical models (14-16) results in the hypertrophy of skeletal muscle fibers and a decrease in adipose tissue mass. Additional preclinical data suggest an anabolic effect on other tissues, including cardiomyocytes (6, 17, 18).

While the role of myostatin and other ActRII ligands as negative regulators of skeletal muscle size is well characterized (4, 8), the effect on cardiac muscle and function is not well understood. Reports of genetic mutations in animals (eg, mstn-/mstn- mouse, Belgian Blue cattle, sheep) suggest conflicting impact on cardiac function (17, 18). In the myostatin null mouse model, the observed cardiac hypertrophy is associated with an increase in left ventricular mass and a larger internal ventricular diameter and volume during systole and diastole that correspond to an enhanced cardiac output (18). The cardiac hypertrophy is not pathological and may be compensatory to the sizeable increase in overall skeletal muscle volume. Cardiac hypertrophy induced by pharmacological inhibition of ActRII in animal models is reversible when the treatment is stopped (Novartis/Eli Lilly – data on file). In mouse models of heart failure, blockade of the ActRII receptor restored or preserved cardiac function (7).

Drugs targeting the myostatin–activin pathway have been explored as treatments for skeletal muscle wasting and metabolic dysfunction (11, 12, 19), and are currently being evaluated (20) to address the corresponding loss of lean mass observed with the GLP-1 weight loss agents. Bimagrumab is a human monoclonal antibody that binds competitively to ActRII with greater affinity than its natural ligands. In preclinical studies, an increase in cardiac size and weight accompanied the large gain in skeletal muscle mass and was reversible with drug withdrawal. Studies of bimagrumab in adult men and women aged 18 to 93 years with acute disuse atrophy (21), sporadic inclusion body myositis (sIBM) (22), chronic obstructive pulmonary disease (COPD) cachexia (23), sarcopenia (11), and obesity with type 2 diabetes (12), have shown significantly increased muscle mass, reduced body fat, and improved metabolic parameters within 8 weeks of exposure that are maintained or further enhanced with repeat dosing. In key clinical studies, echocardiography was used to assess the effects of bimagrumab on myocardial structure and function with up to 52 weeks of drug exposure and showed no adverse effect (22).

Bimagrumab and other drugs targeting anabolic pathways are being studied as synergistic treatments with GLP-1 receptor agonists to offset associated skeletal muscle loss. Preserving skeletal muscle mass and function is particularly important in older populations where rates of obesity and type 2 diabetes are increasing, muscle mass and strength are inherently declining, and many age-associated conditions result in the loss of physical function and capacity. In addition, understanding the effects of chronic drug exposure on the aging heart and cardiovascular parameters is of priority importance. The effect of selectively reducing ActRII signaling on human cardiac structure and function is not well established. Therefore, the present study was undertaken to answer key questions of cardiovascular safety with extended exposure to bimagrumab in healthy older adults.

## Materials and methods

### Study design

**Figure 1:**
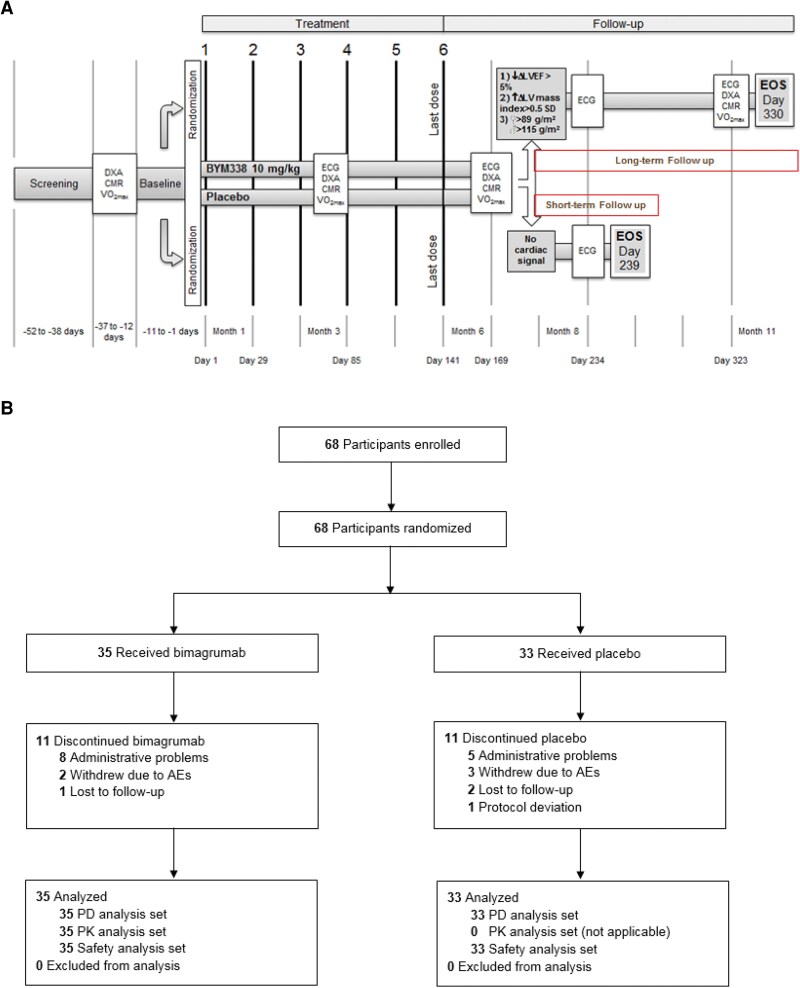
Study design and enrollment. A Study Design. CMR, cardiovascular magnetic resonance; DXA, dual energy X-ray absorptiometry; ECG, electrocardiogram; EOS, end of study; LVEF, left ventricular ejection fraction; LVMI, left ventricular mass index; VO_2_max, maximal oxygen uptake. B Enrollment and Retention. *Administrative problems included travel time to the site, work commitments, and the number and type of assessments, particularly the maximal cardiopulmonary exercise tests. AEs, adverse events; PD, pharmacodynamics; PK, pharmacokinetics.

This 48-week, randomized, double-blind, placebo-controlled study was conducted at a single center in the United States between November 2013 and August 2015. This study included monthly intravenous (IV) dosing over 6 months with bimagrumab 10 mg/kg or placebo, and a safety follow-up period of up to 5 additional months (Fig. 1A). Participants were followed after treatment for 5 months (long term follow-up group) if their left ventricular mass index (LVMI) increased by ≥7.6 g/m^2^ (men) or ≥5.4 g/m^2^ (women), had an overall LVMI ≥115 g/m^2^ (men) or ≥89 g/m^2^ (women), or their left ventricular ejection fraction (LVEF) decreased by >5%. Otherwise, the follow-up period was 2 months (short-term follow-up group).

All study participants provided written informed consent before the beginning of study activities. The study was approved by the site institutional review board and was overseen by an independent data monitoring committee. Study activities were performed in accordance with international guidelines (24, 25) and applicable local regulations.

### Participants

Men and women aged 60 to 86 years with a body mass index (BMI; body weight in kg/height in meters squared) of 18 to 34 kg/m^2^, LVMI (left ventricular mass in grams/body surface area in meters squared) < 115 g/m^2^ (men) and <89 g/m^2^ (women), and LVEF ≥55% were enrolled. Key exclusion criteria included a history of myocardial infarction or the presence of Q waves on resting electrocardiogram (ECG), any medical condition (orthopedic, peripheral vascular disease, unstable angina, restrictive lung disease, or other clinically significant ECG abnormalities) that could interfere with cardiopulmonary exercise testing, or was a contraindication to cardiac magnetic resonance (CMR) imaging.

Participants were randomly assigned in a 1:1 ratio to receive either IV infusion of 10 mg/kg bimagrumab or placebo (dextrose 5% in water) every 4 weeks over 24 weeks. Study site and sponsor personnel were masked to treatment assignment until after database lock.

### Outcomes

Key endpoints included LVMI, LVEF, and other standard cardiovascular variables assessed by cardiac magnetic resonance (CMR), resting and exercise ECG, and Holter monitoring. In addition, markers of cardiac safety and parameters of maximal oxygen uptake (VO_2max_) and body composition (total lean body mass [LBM] and total body fat mass) were measured. Endpoints were assessed in all participants at baseline, 3 months (week 13), and 6 months (week 25) to determine changes from baseline with study treatments.

#### Cardiac imaging assessments

CMR imaging was performed with a 1.5 T Philips Achieva whole body magnetic resonance imaging (MRI) scanner equipped with phased-array body receiver coils. Participants were scanned in the supine position with continuous ECG monitoring to synchronize the imaging acquisition sequences. The imaging protocol consisted of multiplanar scouts collected for localization and a series of sequences performed with an ECG-triggered cine steady-state free precession sequence acquired along the short axis (left atrium and ventricle) to assess cardiac structure and function (26). Additionally, 2- and 4-chamber long-axis image sets and velocity encoded images were acquired to assess the left ventricular inflow and outflow tracts. Cardiac imaging analysis was conducted by an independent central reader (licensed radiologist) and managed by a central imaging vendor that masked treatment and timepoint.

#### Other cardiac parameters

A 12-lead ECG was performed following standard procedure and interpreted by the investigator or another qualified physician.

A 12-lead Holter monitor was used to record continuous, ambulatory ECG data over a 24-hour period during the days when participants received study drug; recording was started before dosing. Recordings were reviewed and interpreted by a vendor-based board-certified cardiologist.

A cardiopulmonary exercise test (27) using a modified Naughton protocol assessed participants' maximum oxygen uptake (VO_2max_) as a measure of cardiovascular functional capacity. Tests were performed at screening to confirm qualification, at weeks 13 and 25, and at week 48 for participants in the longer follow-up group.

#### Body composition

Lean body mass was used as a proxy for skeletal muscle mass. Whole-body dual energy X-ray absorptiometry (DXA) was used to determine changes in LBM and FM over the study period, as in previous studies with bimagrumab (11, 12, 23). DXA scans were analyzed by an independent imaging vendor. Body weights of study participants with values for baseline, 3 and 6 months (EOS) were used to assess the net effect of body composition changes in participants who completed the study treatment regimen.

#### Safety

Safety was assessed throughout the study using the incidence of adverse events (AEs), physical examination, vital signs, and laboratory values for hematology, urinalysis, and clinical chemistry. Additionally, soluble markers of cardiac safety, such as creatine kinase (CK; including CK-MB), N-terminal prohormone of brain natriuretic peptide (NT-pro BNP), and troponin-T, were assessed monthly throughout the study.

#### Statistical analysis

The change from baseline in LVMI and LVEF was analyzed by repeated measures ANCOVA using treatment, visit, treatment*visit, and baseline covariate as fixed effects. The study was powered using a two-sided confidence interval for the bimagrumab/placebo LVEF ratio with a half-width no larger than 6.9% at 6 months, assuming 40 completers. A saturated covariance structure was used for observations within the same participant. Similarly, log-transformed changes from baseline of LBM and FM were analyzed considering log baseline as a covariate. Lean body mass results were back-transformed. Electrocardiogram parameters for heart rate and Fridericia-corrected QT interval values (QTcF) were analyzed at each visit using treatment, timepoint, treatment*timepoint, and baseline covariate as fixed effects. The point estimate and 90% confidence interval (CI) for the treatment comparison (bimagrumab vs placebo) were provided.

## Results

Sixty-eight participants were randomly assigned to either bimagrumab (*n* = 35, 13 men and 22 women) or placebo (*n* = 33, 13 men and 20 women; Table 1). Forty-nine participants (72.1%; bimagrumab, *n* = 25; placebo, *n* = 24) completed the 6-month treatment, and 46 participants (67.6%; bimagrumab, *n* = 24; placebo, *n* = 22) completed the study. Twenty-two participants discontinued (Fig. 1B), largely due to the required repeated maximal cardiopulmonary exercise tests, work conflicts, and moving out of the area (*n* = 13). Demographic characteristics (Table 1) and medical histories of the study participants were similar in both treatment arms. Treatment adherence was ensured by clinic-based administration of the study drug and confirmed by pharmacokinetic assessment.

**Table 1:** Summary of participant demographics at baseline

	Bimagrumab (*n* = 35)	Placebo (*n* = 33)	Total (*N* = 68)
Age, years	67.8 (5.6)	67.8 (6.6)	67.8 (6.1)
Gender, n (%)			
Women	22 (63)	20 (61)	42 (62)
Men	13 (37)	13 (39)	26 (38)
Race, n (%)			
Caucasian	32 (91)	30 (91)	62 (91)
Black	3 (9)	2 (6)	5 (7)
Other	0 (0)	1 (3)	1 (1)
Ethnicity, n (%)			
Hispanic/Latino	35 (100)	31 (94)	66 (97)
Other	0 (0)	2 (6)	2 (3)
Height, cm	160.4 (8.7)	160.8 (8.9)	160.6 (8.8)
Weight, kg	70.5 (10.2)	69.2 (10.9)	69.8 (10.5)
BMI, kg/m^2^	27.4 (3.4)	26.8 (3.7)	27.1 (3.6)

Values are presented in mean (SD), unless specified.

Abbreviations: BMI, body mass index is body weight in kg per height in meters squared; SD, standard deviation.

**Figure 2:**
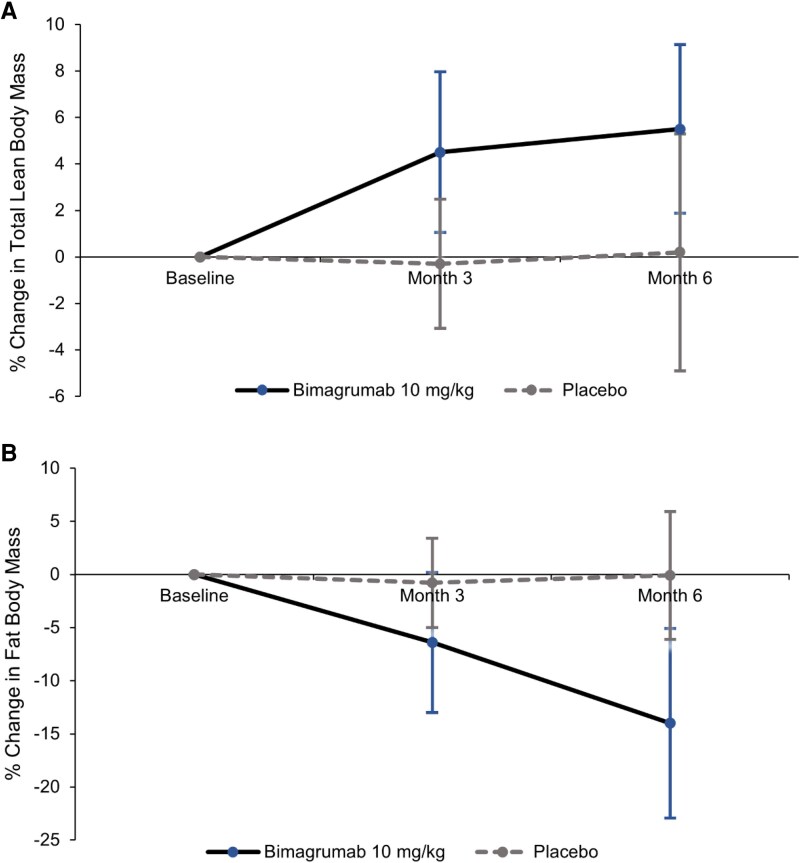
Percent change in body composition. A Lean Body Mass. Analysis included the entire study population. Difference from baseline at months 3 and 6, both *P* < .001. B Body Fat Mass. Analysis included the entire study population. Difference from baseline at months 3 and 6, both *P* < .001.

As anticipated, total LBM increased over the 6-month treatment period in the bimagrumab group, while it remained constant in the placebo group (Table 2). In the bimagrumab group, total LBM (mean [SD]) increased from baseline by 4.5 [3.5] % at 3 months and 5.5 [3.6] % at 6 months (both *P* < .001) (Fig. 2A), which corresponded to gains of 1.6 [1.2] kg and 2.0 [1.4] kg, respectively. Conversely, total body fat mass in the bimagrumab-treated group declined from baseline by −6.4 [6.9] % at 3 months and −14.0 [8.9] % at 6 months (both *P* < .001) (Fig. 2B), resulting in corresponding changes in fat mass of −1.6 [1.7] kg at 3 months and −3.5 [2.1] kg at 6 months. In contrast, no change in fat mass was observed in the placebo-treated group (Table 2). Body weights of participants with measurements at baseline, 3, and 6 months of treatment (*n* = 25 bimagrumab; *n* = 27 placebo) showed little change in either group over 3 months. However, at 6 months, the bimagrumab group showed a net loss of −1.7 kg while the placebo group had a gain of 1.0 kg (Table 2). The between-treatment difference of −2.64 kg (95% CI −4.11, −1.17) at 6 months was statistically significant (*P* < .001).

**Table 2:** Changes in body composition, weight and cardiac mass and function

	Bimagrumab	Placebo
**LBM, kg/m^2^** *^a^*		
Baseline	37.54 (7.77)	37.77 (7.29)
3 months	39.55 (7.33)	37.27 (7.28)
6 months	40.51 (7.61)	37.23 (7.19)
**Body fat mass, kg/m^2^** *^a^*		
Baseline	26.38 (6.94)	25.19 (7.12)
3 months	25.49 (7.53)	24.63 (7.59)
6 months	23.59 (7.90)	24.62 (8.16)
**Body weight, kg** *^b^*		
Baseline	72.42 (8.86)	68.72 (11.54)
3 months	72.69 (9.20)	69.03 (11.87)
6 months	70.77 (10.15)	69.72 (12.01)
**LVMI, g/m^2,^** *^c^*		
Baseline	39.2 (9.3)	40.3 (7.4)
3 months	37.5 (8.9)	38.5 (7.4)
6 months	37.4 (10.2)	36.8 (5.1)
**LVEF, %**		
Baseline	63.6 (4.7)	63.2 (5.2)
3 months	63.7 (5.4)	61.9 (6.2)
6 months	62 (5.3)	59.9 (5.0)
**LVESVI, mL/m^2^**		
Baseline	27.2 (5.3)	27.9 (5.7)
3 months	26.9 (6.8)	26.3 (5.6)
6 months	26.5 (6.8)	29.9 (6.2)
**LVEDVI, mL/m^2^**		
Baseline	74.7 (11.4)	75.8 (9.1)
3 months	73.9 (14.0)	69.1 (8.7)
6 months	70.2 (15.4)	74.3 (10.7)
**Cardiac output, L/min**		
Baseline	5.2 (1.1)	5.5 (1.2)
3 months	5.2 (1.1)	4.9 (1.1)
6 months	4.9 (0.9)	5 (1.2)
**Cardiac index, L/min/m^2^**		
Baseline	3 (0.6)	3.2 (0.6)
3 months	3 (0.6)	2.8 (0.5)
6 months	2.8 (0.6)	2.9 (0.7)

Values presented as mean (SD) unless otherwise specified.

Abbreviations: CMR, cardiac magnetic resonance; DXA, dual energy X-ray absorptiometry; LVEDVI, left ventricular end-diastolic volume index; LVESVI, left ventricular end-systolic volume index; LVEF, left ventricular ejection fraction; LVMI, left ventricular mass index; SD, standard deviation.

^*a*^Units are per m^2^ of height assessed by DXA.

^*b*^Bodyweight data from 52 participants with values at baseline, 3 and 6 months.

^*c*^Units are per m^2^ body surface area (BSA) assessed by CMR.

**Figure 3:**
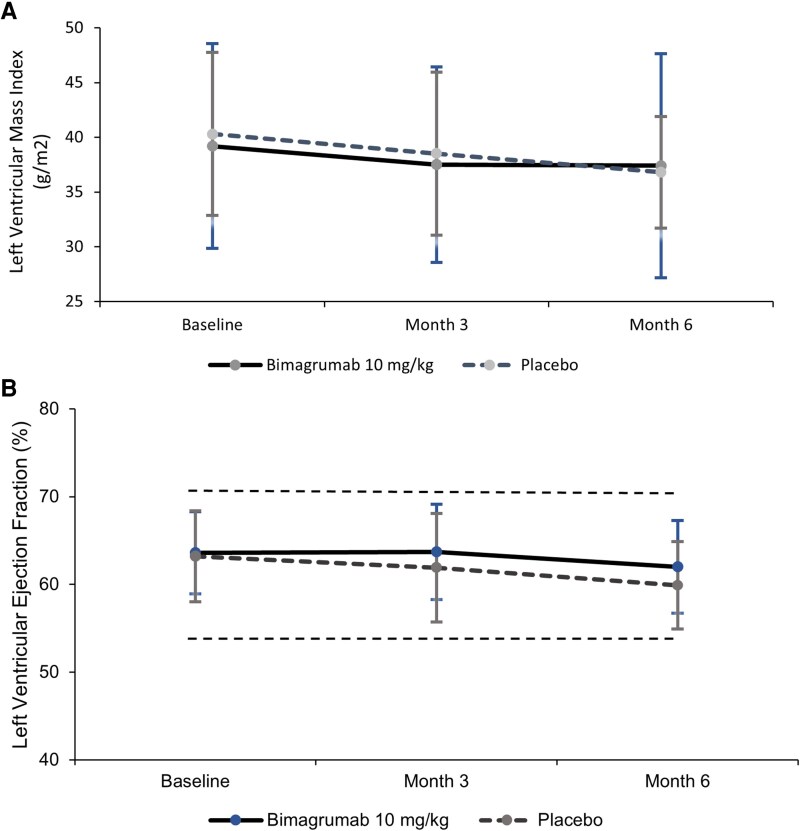
Change in cardiac magnetic resonance parameters over 6 months of treatment (mean ± SD). A Left Ventricular Mass Index, B Left Ventricular Ejection Fraction.

Decreases in mean left ventricular mass index (LVMI) were observed in both the bimagrumab and placebo groups from baseline (mean [SD] at 3 months (−1.55 [3.61] g/m^2^ vs −2.15 [4.45] g/m^2^) and 6 months (−2.26 [3.07] g/m^2^ vs −4.01 [4.74] g/m^2^)) (Table 3 and Fig. 3A). The reductions in LVMI observed at 6 months did not differ by treatment (*P* = .148) (Table 2). On average, the change in wall thickness for any segment did not exceed 6 mm at 6 months or follow-up timepoints (data not shown). The LVMI and segmental wall thickness changes were judged not clinically relevant, and therefore, no participant met the criterion for long-term follow-up based on these parameters.

**Table 3:** Absolute change from baseline in left ventricular mass index and left ventricular ejection fraction

Visit		Treatment		
		Bimagrumab 10 mg/kg	Placebo	LS mean difference (90% CI) *P* value
**LVMI (g/m^2^)**				
3 months (week 13)	n Mean (SD)	27 −1.55 (3.61)	28 −2.15 (4.45)	0.33 (−1.37, 2.03), 0.748
6 months (week 25)	n Mean (SD)	25 −2.26 (3.07)	24 −4.01 (4.74)	1.58 (−0.22, 3.38) 0.148
**LVEF (%)**				
3 months (week 13)	n Mean (SD)	27 0.73 (6.17)	28 −1.44 (4.91)	1.94 (−0.32, 4.21) 0.158
6 months (week 25)	n Mean (SD)	25 −0.86 (5.35)	24 −2.67 (5.72)	1.97 (−0.43, 4.37) 0.176

Analysis included entire study population.

Abbreviations: CI, confidence interval; LS, least square mean; SD, standard deviation.

Horizontal dotted line represents the upper and lower limit of normal LVEF values.

Changes in left ventricular ejection fraction (LVEF) within and between groups were not clinically relevant or statistically significant at any timepoint (Tables 2 and 3; Fig. 3B). LVEF in the bimagrumab- and placebo-treated groups were comparable at baseline (mean [SD]: 63.6 [4.7] vs 63.2 [5.2] %), 3 months (63.7 [5.4] % vs 61.9 [6.2] %), and at 6 months (62.0 [5.3] % vs 59.9 [5.0] %) with all participants remaining within normal range (ie, 50-70%) (Table 2). Participants receiving bimagrumab had a mean increase in LVEF of 0.73% at 3 months, followed by a decrease of −0.86% at 6 months, compared to baseline. Participants receiving placebo had mean decreases of −1.44% and −2.67% at 3 months and 6 months, respectively (Table 2). Based on changes in LVEF over the study, 13 patients entered long-term follow-up, while 36 entered short-term follow-up. Those administered bimagrumab who completed the short-term follow-up (*n* = 20) showed mean increases in LVEF from 62.1% at baseline to 64.2% (90% CI: 3.15 [0.57, 5.73]) at 3 months and 63.1% (90% CI: 1.64 [−0.94, 4.22]) at 6 months. These changes were not statistically significant. The placebo group showed no change from 60.9% at baseline to 60.5% at 3 months and 60.9% at 6 months.

Thirteen participants qualified for the long-term follow-up arm of the study because their LVEF decreased by >5% (bimagrumab, *n* = 5; placebo, *n* = 8) with a decrease in mean LVEF in the bimagrumab subgroup from 65.8% at baseline to 61.3% at 3 months, 57.8% at 6 months, and 59.2% at 12 months. The placebo subgroup had comparable decreases from 66.0% at baseline to 62.0% at 3 months, 58.0% at 6 months, and 59.3% at 12 months. No statistical difference between treatment groups was seen at any timepoint. At no time did any participant's LVEF drop below the lower limit of normal of 50%.

Similarly, no differences were seen between treatments in other measures of cardiovascular function. Evaluation of cardiac index, resting and exercise heart rate, all cardiopulmonary exercise testing parameters, systolic and diastolic blood pressure, and left ventricular end-systolic and end-diastolic volume indices showed no effect of bimagrumab when compared to placebo (Tables 2 and  4).

**Table 4:** Changes in cardiovascular physiology

	Bimagrumab	Placebo
**Resting heart rate, beats/min**		
Baseline	63.5 (7.4)	66.6 (9.8)
3 months	64 (5.9)	66.5 (10.4)
6 months	64.8 (8.1)	65.6 (11.7)
**Systolic/diastolic BP, mm Hg—supine**		
Baseline	127.8 (15.4)/74.2 (6.2)	124.6 (16.3)/74.4 (8.7)
3 months	123.5 (15.3)/72.6 (7.5)	118.1 (11.6)/73.4 (7.9)
6 months	127.2 (15.2)/74.1 (6.2)	125.1 (15.9)/75.5 (7.9)
**Systolic/diastolic BP, mm Hg—standing**		
Baseline	132.5 (14.1)/80.1 (6.1)	128.2 (14.0)/79.8 (7.2)
3 months	130.1 (18.6)/78.3 (6.6)	124.4 (12.9)/77.6 (9.1)
6 months	130.9 (13.1)/78 (5.1)	128.3 (13.8)/78.7 (6.7)
**Exercise test duration, min**		
Baseline	14.2 (4.8)	13.8 (2.9)
3 months	12.9 (4.6)	12.6 (3.2)
6 months	12.5 (4.9)	12.8 (2.7)
**VO_2max_ (mL O_2_/[kg × min])**		
Baseline	22.2 (4.8)	21.4 (5.1)
3 months	21.5 (7.1)	20.8 (4.8)
6 months	20.8 (6.5)	20.3 (4.8)

Values presented as mean (SD) unless otherwise specified.

Abbreviations: BP, blood pressure; SD, standard deviation; VO_2max_, maximal oxygen uptake.

### Drug exposure and safety

As expected, the pharmacokinetic profile of bimagrumab in the healthy participants declined in a nonlinear fashion consistent with target-mediated drug disposition typical of monoclonal antibodies, as described in prior studies with bimagrumab (22, 28). No immunogenicity signal was reported in participants receiving at least 1 dose of bimagrumab.

The majority of AEs were of mild or moderate severity. Muscle spasms, described as transient, involuntary, generally painless muscle contractions or fasciculations, were observed more often in the bimagrumab group (57.1% vs placebo 6.1%), which was consistent with prior studies (11, 22, 23). No new safety signal was reported, and no clinically relevant changes were seen in any of the serum cardiac safety biomarkers from either treatment group (Tables 5 and 6).

**Table 5:** Frequency of reported adverse events

Summary of AEs*^a^*	Bimagrumab (*n* = 35)	Placebo (*n* = 33)	Total (*N* = 68)
Participants with AE(s)	31 (88.6)	19 (57.6)	50 (73.5)
Muscle spasms	20 (57.1)	2 (6.1)	22 (32.4)
Headache	8 (22.9)	3 (9.1)	11 (16.2)
Influenza-like illness	6 (17.1)	5 (15.2)	11 (16.2)
Dizziness	5 (14.3)	4 (12.1)	9 (13.2)
Pain in extremity	4 (11.4)	3 (9.1)	7 (10.3)
Back pain	5 (14.3)	1 (3.0)	6 (8.8)
Fatigue	2 (5.7)	4 (12.1)	6 (8.8)
Acne	5 (14.3)	0 (0)	5 (7.4)
Arthralgia	3 (8.6)	2 (6.1)	5 (7.4)
Diarrhea	3 (8.6)	2 (6.1)	5 (7.4)
Abdominal pain	2 (5.7)	2 (6.1)	4 (5.9)
Cough	0 (0)	4 (12.1)	4 (5.9)
Lipase increased	3 (8.6)	1 (3.0)	4 (5.9)
Nausea	3 (8.6)	1 (3.0)	4 (5.9)
Neck pain	3 (8.6)	1 (3.0)	4 (5.9)

Abbreviation: AEs, adverse events.

^*a*^AEs ≥5% in total population have been listed. Data presented as n (%).

**Table 6:** Changes in cardiac safety biomarkers

	Bimagrumab	Placebo
**Creatinine kinase, ukat/L**		
Screening	1.9 (0.7)	1.8 (0.9)
3 months	3.2 (1.6)	1.9 (1.2)
6 months	3.7 (2.8)	1.4 (0.6)
**Creatinine kinase–MB, μg/L**		
Screening	1.6 (1.3)	1.6 (1.2)
3 months	2.0 (1.2)	1.9 (1.6)
6 months	2.0 (1.0)	1.4 (1.1)
**N-terminal prohormone of brain natriuretic peptide, pmol/L**		
Screening	8.4 (5.3)	11.6 (12.4)
3 months	10.4 (6.2)	13.3 (19.0)
6 months	10.8 (7.1)	12 (10.9)
**Troponin T, μg/L**		
Screening	0 (0)	0 (0)
3 months	0 (0)	0 (0)
6 months	0 (0)	0 (0)

Values presented as mean (SD) unless otherwise specified.

Abbreviation: SD, standard deviation.

## Discussion

In this study, 6 months of myostatin–activin pathway inhibition with bimagrumab resulted in a significant measurable increase in LBM and decrease in fat mass with no adverse effects on cardiac structure or hemodynamic function, as measured by a change in LVMI, left ventricular wall thickness, LVEF, resting cardiac index, VO_2_max, ECG, resting or exercise heart rate, and blood pressure. In addition, there was no effect on serum biomarkers of cardiac injury. These findings, determined by cardiac magnetic resonance imaging, Holter monitor, cardiopulmonary exercise testing, and cardiac safety biomarkers, provide a comprehensive look at the effects of extended inhibition of the myostatin–activin pathway on the human heart and cardiovascular physiology, supporting prior data to suggest that up to 6 months of bimagrumab treatment can be safe for healthy older adults.

The absence of a treatment effect on LVMI, left ventricular wall thickness, and LVEF suggests that the bimagrumab-induced increase in skeletal muscle mass did not lead to cardiac hypertrophy and had no effect on cardiac function in this older adult population. Animal studies have shown mixed results. Our findings are in contrast to animal studies across species from mice to cattle, where skeletal muscle anabolism through the genetic deletion or inhibition of ActRII ligands (ie, myostatin) resulted in a hypertrophic effect 3- to 8-fold greater than that typically observed in humans (15-40% increase in whole-body lean mass vs 5-8%), with much lower effect on fat mass in non-obese animals (8, 16, 29). The cardiac hypertrophy seen in these animals was not pathological and was probably an adaptive physiological response to the greater oxygen needs of the increased skeletal muscle mass. This hypothesis of a physiological adaptation to a large increase in muscle mass is supported by the similar body weight/heart weight ratio seen in myostatin null mice and wild-type littermates, as well as the comparable—and importantly, reversible upon cessation of treatment—cardiac and skeletal muscle changes seen in wild-type mice and rats administered ActRII receptor or myostatin blockers (16, 18). Therefore, the lack of cardiac structural or physiological changes observed in the current study was likely due to the relatively small increase in LBM seen in humans being below a cardiac muscle hypertrophic response threshold.

In addition to the more established effects on skeletal muscle, animal data suggest a potential benefit of anti-myostatin and myostatin–activin pathway inhibition on cardiovascular function. Myostatin, a potent myokine that negatively regulates skeletal muscle growth, is also expressed in cardiomyocytes (5, 6). Data from myostatin null mice show an increase in cardiac output with associated hypertrophy (18). Butcher and colleagues (6) reported an increased fractional shortening in the myostatin null mouse, with no effect on cardiac size, heart rate, or blood pressure. Data in myostatin null rats also suggests a role for myostatin in reducing pressure overload-induced cardiac hypertrophy (30). A similar antihypertrophic response was observed in wild-type mice with induced cardiac ischemia, where an increase in myostatin originating from myocardium was seen (31). Further, increased cardiac ActRII signaling impairs cardiac function in animal models of heart failure, while decreased signaling via pharmacologic inhibition or genetic deletion of ligands preserves or restores cardiac function (7). Whether inhibition of myostatin–activin pathway signaling can improve cardiac function in humans with pathologic dysfunction remains to be determined. However, the data presented here suggest that pharmacological interventions working through the myostatin–activin pathway may be safe to explore in patients with heart failure.

Bimagrumab works as a receptor antagonist blocking the activity of all ligands of ActRII (ie, myostatin/GDF-8, Activin A, and GDF-11), while maintaining the availability of the ligands in circulation to act through other receptors. Consistent with previous studies in individuals with age-related sarcopenia (11), acute disuse atrophy (21), sporadic inclusion body myositis (22), chronic obstructive pulmonary disease cachexia (23), and obesity with type 2 diabetes (12), bimagrumab increased LBM and decreased total body FM starting with the first dose and continuing throughout the treatment period in this healthy older population. Although this study was limited to six months of drug treatment, it is important to note that LBM increases plateaus after approximately two months of treatment in humans, so the results at 6 months reflect steady state on somatic lean mass increase due to bimagrumab. In contrast, fat mass continues to decline for at least 12 months with bimagrumab treatment, but further reduction in body fat is not expected to compromise cardiac function (12). Combined with the aggregated safety data across studies, these body composition data confirm the generally safe, consistent, predictable pharmacodynamic efficacy of bimagrumab.

This study had limitations. This was a small study performed at a single site with a relatively homogeneous population of healthy older adults, which may limit the generalizability of the findings. In addition, the baseline values for LVMI were lower than expected but still within the normal range based on earlier data (32) and more recent cardiac MRI reference ranges established in the UK Biobank for healthy elderly (33). The lower baseline LVMI may be in part due to the somewhat shorter stature of this predominantly Hispanic American study population in South Florida. The MRI methodology used in this study provided a more accurate and precise measure of structural and functional changes than the echocardiography used in earlier studies with up to 52 weeks of bimagrumab treatment (22). However, larger studies with bimagrumab of similar and longer duration of treatment are needed to further support these current findings.

## Conclusions

Six months of treatment with bimagrumab, an ActRII receptor antagonist leading to inhibition of the myostatin–activin pathway, had no adverse effect on cardiac structure or function, nor on resting heart rate or blood pressure of healthy older adults. Consistent with its established safety profile, bimagrumab was safe and well tolerated with no cardiovascular or other safety concerns reported. The paucity of clinical cardiac safety data for drugs targeting the myostatin–activin pathway has limited the inclusion in clinical trials of patients with certain cardiovascular risk factors and conditions. These data support further study of targeting the pathway to treat various conditions in older adults with and without cardiovascular disease. Bimagrumab, with its positive safety profile and predictable effect on skeletal muscle growth and adipose tissue loss, may be a suitable solution to the unwanted consequence of skeletal muscle loss seen with GLP-1 receptor agonist use.

## Data Availability

The datasets used and/or analyzed during the study are available from the corresponding author to qualified investigators with legitimate purposes.
